# Case report: Short-term efficacy and changes in ^18^F-FDG-PET with acute multi-target stimulation in spinocerebellar ataxia type 3 (SCA3/MJD)

**DOI:** 10.3389/fneur.2023.1246430

**Published:** 2023-09-27

**Authors:** Zhiqiang Cui, Yina Lan, Yan Chang, Xinyun Liu, Jian Wang, Xin Lou, Ruimin Wang

**Affiliations:** ^1^Department of Neurosurgery, Chinese People's Liberation Army (PLA) General Hospital, Beijing, China; ^2^Department of Radiology, Chinese People's Liberation Army (PLA) General Hospital, Beijing, China; ^3^Department of Nuclear Medicine, Chinese People's Liberation Army (PLA) General Hospital, Beijing, China

**Keywords:** spinocerebellar ataxia type 3 (SCA3), Machado-Joseph disease (MJD), deep brain stimulation (DBS), dentate nucleus (DN), globus pallidus internus (GPi), ^18^F-fluoro-2-deoxy-d-glucose positron emission computed tomography (^18^F-FDG-PET), dopamine transporter (DAT) PET

## Abstract

**Objective:**

Spinocerebellar ataxia type 3 (SCA3), also known as Machado-Joseph disease (MJD), is a rare neurodegenerative disease for which there is no specific treatment. Very few cases have been treated with single-target deep brain stimulation (DBS), and the results were not satisfactory. We applied multi-target DBS to an SCA3/MJD patient and performed positron emission computed tomography (PET) before and after DBS to explore the short-term clinical therapeutic effect.

**Materials and methods:**

A 26-year-old right-hand-dominant female with a family history of SCA3/MJD suffered from cerebellar ataxia and dystonia. Genetic testing indicated an expanded CAG trinucleotide repeat in the *ATXN3* gene and a diagnosis of SCA3/MJD. Conservative treatment had no obvious effect; therefore, leads were implanted in the bilateral dentate nucleus (DN) and the globus pallidus internus (GPi) and connected to an external stimulation device. The treatment effect was evaluated in a double-blind, randomized protocol in five phases (over a total of 15 days): no stimulation, GPi, DN, or sham stimulation, and combined GPi and DN stimulation. ^18^F-fluoro-2-deoxy-d-glucose and dopamine transporter PET, Scale for the Assessment and Rating of Ataxia, Fahn-Tolosa-Marin Clinical Rating Scale for Tremor (FTM), Burke-Fahn-Marsden Dystonia Rating Scale (BFMDRS), and SF-36 quality of life scores were compared before and after DBS.

**Results:**

The Total Scale for the Assessment and Rating of Ataxia scores improved by ~42% (from 24 to 14). The BFMDRS movement scores improved by ~30% (from 40.5 to 28.5). The BFMDRS disability scores improved by ~12.5% (from 16 to 14). Daily living activities were not noticeably improved. Compared with the findings in pre-DBS imaging, ^18^F-fluoro-2-deoxy-d-glucose uptake increased in the cerebellum, while according to dopamine transporter imaging, there were no significant differences in the bilateral caudate nucleus and putamen.

**Conclusion:**

Multi-target acute stimulation (DN DBS and GPi DBS) in SCA3/MJD can mildly improve cerebellar ataxia and dystonia and increase cerebellar metabolism.

## 1. Introduction

Spinocerebellar ataxia type 3 (SCA3), also known as Machado-Joseph disease (MJD), is a rare neurodegenerative disease characterized by progressive cerebellar ataxia and variable symptoms, including pyramidal signs, a dystonic-rigid extrapyramidal syndrome, significant peripheral amyotrophy and generalized areflexia, progressive external ophthalmoplegia, action-induced facial and lingual fasciculations, and bulging eyes (1). SCA3/MJD is caused by a CAG trinucleotide repeat expansion in exon 10 of the *ATXN3* gene on chromosome 14(p32). The core clinical feature of SCA3/MJD is progressive ataxia resulting from cerebellar and brainstem dysfunction. There is no specific treatment for SCA3/MJD, and treatment goals are to maximize function and reduce complications. It is important to remember, however, that numerous symptoms occurring in SCA3/MJD can respond to symptomatic therapy (2).

Deep brain stimulation (DBS) has been used to treat various symptoms in patients suffering from movement disorders, such as Parkinson's disease, dystonia, and essential tremor. Though ataxia syndromes have not been formally addressed with DBS, patients with ataxia, tremor, or dystonia have been administered DBS in different targets, resulting in partial relief of symptoms. Studies reporting treatment of SCA3/MJD with DBS have targeted the thalamus and cerebellum (3–8). These treatments were single-target stimulations, and the results were not very satisfactory. Although some results were statistically significant, no case showed an improvement of more than 50%. Some reports show no significant difference in Scale for the Assessment and Rating of Ataxia (SARA) scores before and after treatment. It is still unknown whether multi-target stimulation can have an improved therapeutic effect on SCA3/MJD.

Here, we tested whether bilateral dentate nucleus (DN) and globus pallidus internus (GPi) modulation can reduce SCA3/MJD symptoms of cerebellar ataxia and dystonia in a sham-controlled, double-blind study (*n* = 1).

## 2. Methods

### 2.1. Patient

The patient was a 26-year-old right-hand-dominant female with a history of a slight bilateral hand tremor, balance disorder, and gait abnormality since the age of 15 years. She had a family history of SCA3/MJD, with her mother and grandfather affected. Genetic testing revealed the CAG repeat numbers in the *ATXN3* gene to be 23 and 68, confirming a diagnosis of SCA3/MJD. She took oral medication and received rehabilitation training; however, her symptoms of tremor, balance disorder, and gait abnormality gradually worsened, and new symptoms, such as neck stiffness and facial dystonia, developed in recent years. Low-frequency transcranial magnetic stimulation on the DN has a certain effect, but the effect is short, and symptoms gradually became more aggravated. Therefore, patients and their families seek surgical treatment. After admission, we assessed the severity of the disease using the following scores (see Tables 1–3 for specific scores). The patient mildly reacts to levodopa; therefore, we also conducted a levodopa challenge test, which produced an improvement of 9%.

**Table 1:** SARA scores before and after stimulation.

**SARA (9)**			**None**	**GPi**	**DN**	**“Sham”**	**GPi + DN**
**Day**	**Range**	**Baseline**	**0–3**	**4**	**7**	**10**	**13–15**
Gait	0–8	7	6 (0.14)	6 (0.14)	4 (0.43)	6 (0.14)	4 (0.43)
Stance	0–6	4	4 (0)	4 (0)	2 (0.50)	4 (0)	2 (0.50)
Sitting	0–4	2	2 (0)	2 (0)	2 (0)	2 (0)	1 (0.50)
Speech disturbance	0–6	4	4 (0)	4 (0)	3 (0.25)	4 (0)	3 (0.25)
Finger chase	0–4	1	1 (0)	1 (0)	1 (0)	1 (0)	1 (0)
Nose-finger test	0–4	2	2 (0)	2 (0)	1 (0.50)	2 (0)	1 (0.50)
Fast alternating hand movements	0–4	2	2 (0)	2 (0)	1 (0)	2 (0)	1 (0.50)
Heel-shin slide	0–4	3	2 (0.33)	2 (0.33)	1 (0.67)	3 (0)	1 (0.67)
Total score	0–40	24	23 (0.04)	23 (0.04)	15 (0.38)	23 (0.04)	14 (0.42)

SARA, Scale for the Assessment and Rating of Ataxia; GPi, globus pallidus internus; DN, dentate nucleus.

**Table 2:** BFMDRS movement scores before and after stimulation.

			**None**	**GPi**	**DN**	**“Sham”**	**GPi + DN**
**BFMDRS movement** **(****10****)**							
Day	Range	Baseline	0–3	4	7	10	13–15
Eye	0–8	6	4.5 (0.25)	3 (0.50)	6 (0)	6 (0)	3 (0.50)
Mouth	0–8	4.5	4.5 (0)	2 (0.56)	4.5 (0)	4.5 (0)	2 (0.56)
Speech and swallowing	0–16	8	8 (0)	8 (0)	8 (0)	8 (0)	8 (0)
Neck	0–8	1	1 (0)	0.5 (0.50)	1 (0)	1 (0)	0.5
R arm	0–16	1	1 (0)	1 (0)	1 (0)	1 (0)	1 (0)
L arm	0–16	1	1 (0)	1 (0)	1 (0)	1 (0)	1 (0)
Trunk	0–16	1	1 (0)	1 (0)	1 (0)	1 (0)	1 (0)
R leg	0–16	9	9 (0)	6 (0.33)	9 (0)	9 (0)	6 (0.33)
L leg	0–16	9	9 (0)	6 (0.33)	9 (0)	9 (0)	6 (0.33)
Total score	0–120	40.5	39 (0.06)	28.5 (0.30)	40.5 (0)	40.5 (0)	28.5 (0.30)
**BFMDRS disability** **(****10****)**							
Speech	0–4	3	3 (0)	3 (0)	3 (0)	3 (0)	3 (0)
Writing	0–4	1	1 (0)	1 (0)	1 (0)	1 (0)	1 (0)
Feeding	0–4	1	1 (0)	1 (0)	1 (0)	1 (0)	1 (0)
Eating and swallowing	0–4	1	1 (0)	1 (0)	1 (0)	1 (0)	1 (0)
Hygiene	0–4	2	2 (0)	2 (0)	2 (0)	2 (0)	2 (0)
Dressing	0–4	2	2 (0)	2 (0)	2 (0)	2 (0)	2 (0)
Walking	0–6	6	6 (0)	4 (0.33)	6 (0)	6 (0)	4 (0.33)
Total score	0–30	16	16 (0)	14 (0.125)	16 (0)	16 (0)	14 (0.125)

BFMDRS, Burke-Fahn-Marsden Dystonia Rating Scale; GPi, globus pallidus internus; DN, dentate nucleus.

**Table 3:** Other characteristics before and after stimulation.

**Other scores**	**Range**	**Before surgery**	**GPI + DN stimulation**
FTM (16)	0–144	29	25
SF-36 (17)	0–146	64	69
ADL (18)	0–56	36	36
MMSE (19)	0–30	28	28
MoCA (20)	0–30	28	28
HAMA (21)	0–56	9	11
HAMD (22)	0–76	8	8

FTM, the Fahn-Tolosa-Marin Clinical Rating Scale for Tremor; SF-36, SF-36 quality of life measurements; ADL, activities of daily living; MMSE, the Mini-Mental State Examination; MoCA, the Montreal Cognitive Assessment; HAMA, the Hamilton Anxiety Scale; HAMD, the Hamilton Depression Scale.

### 2.2. Magnetic resonance imaging

**Figure 1:**
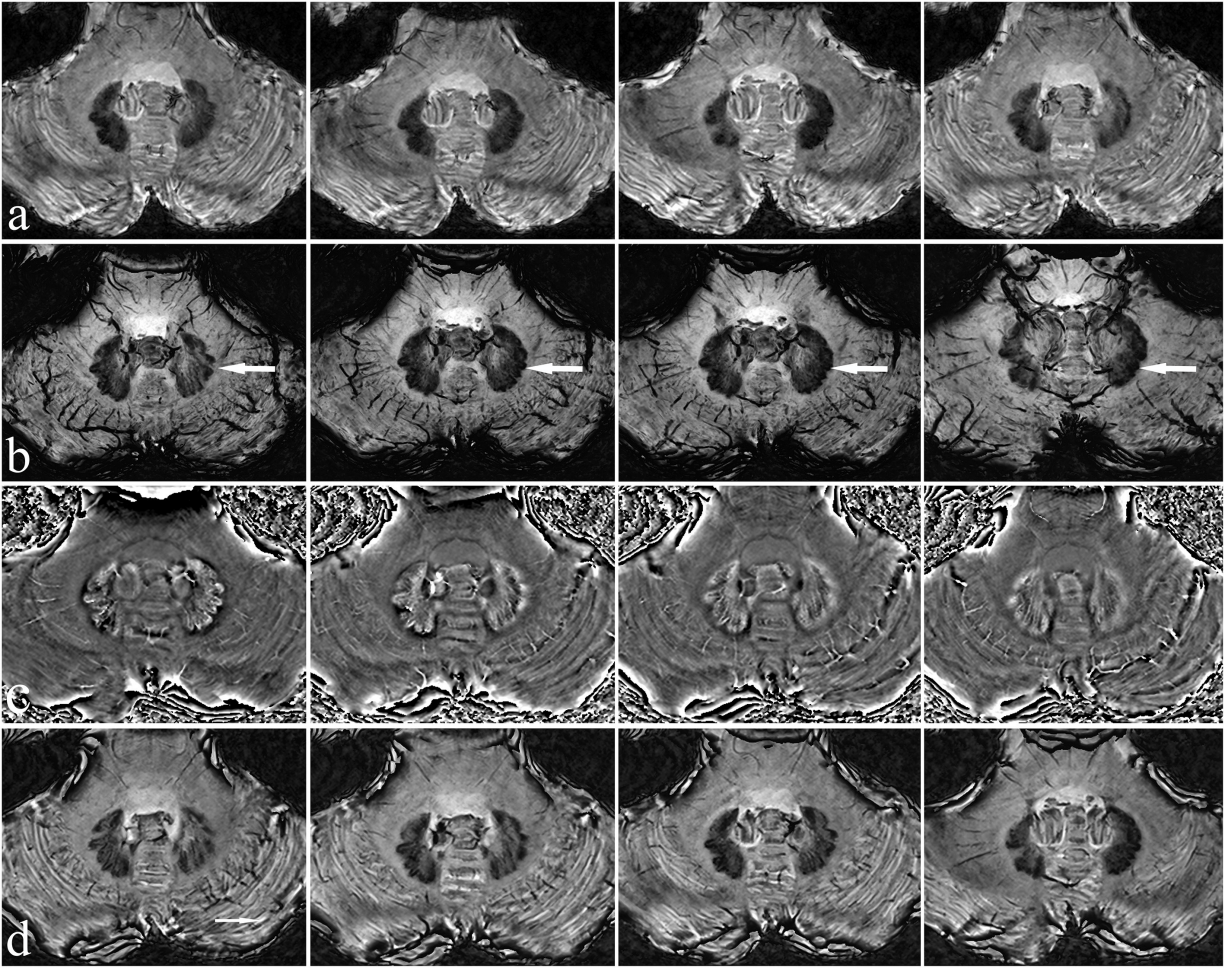
Characteristics of the patient's dentate nucleus on 7T MRI. There is a clear view of the dentate nucleus of the cerebellum. Mag **(a)**, Mip **(b)**, Pha **(c)**, and SWI images **(d)** have different characteristics, with Mip images being the clearest (see arrow).

A 7T magnetic resonance imaging (MRI) scan (Siemens Healthcare, Erlangen, Germany) was first performed on the patient to examine the morphology and structure of the cerebellum and DN in greater detail. After processing, magnitude (Mag), phase (Pha), minimum-intensity projection (Mip), and susceptibility weighted images (SWI) were obtained (Figure 1).

### 2.3. The ^18^F-fluoro-2-deoxy-d-glucose images and dopamine transporter imaging

**Figure 2:**
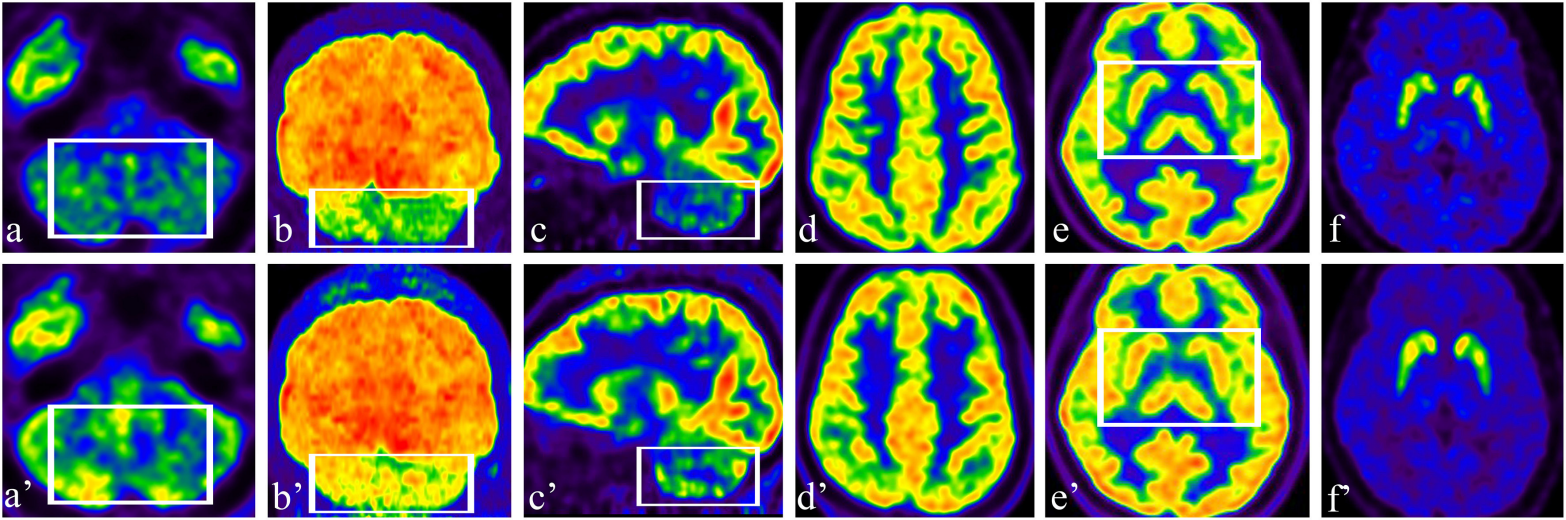
**(a–c)** Preoperative axial, coronal, and sagittal ^18^F-FDG images showed hypometabolism in the bilateral cerebellar hemisphere; **(a'–c')** Postoperative axial, coronal, and sagittal ^18^F-FDG images showed increased uptake in the cerebellum (see box). **(d, e)** Preoperative axial ^18^F-FDG images showed normal metabolism in the bilateral cerebral cortex, GPi area, and basal ganglia; **(d', e')** There was no change in ^18^F-FDG in the cerebral cortex, GPi area, or basal ganglia after stimulation (see box). **(f)** Preoperative axial DAT PET/CT showed normal DAT density in the bilateral caudate nucleus and putamen; **(f')** Compared with the findings in a pre-stimulation image, there were no significant differences in DAT binding in the bilateral caudate nucleus and putamen.

Brain glucose metabolism was assessed by ^18^F-FDG and DAT positron emission tomography/computed tomography (PET/CT) imaging using ^11^C-labeled 2-beta-carbomethoxy-3beta-(4-fluorophenyl) tropane (^11^C-β-CFT) and a Siemens Advance PET scanner (Biography Vision, Siemens Healthcare, Erlangen, Germany). A 5 mCi (185 MBq) of ^11^C-β-CFT was injected intravenously, and images were acquired in the three-dimensional mode at 120 KVp and 380 mAs during a 10-min session. Patients fasted overnight for 6 h before FDG-PET. Patients were injected with 370 MBq of ^18^F-FDG, in a dimly lit room with minimal background noise. Fifty minutes after ^18^F-FDG injection, a scan lasting 10 min was acquired (Figures 2a–e).

### 2.4. The DN pathway and the design of its targeting

**Figure 3:**
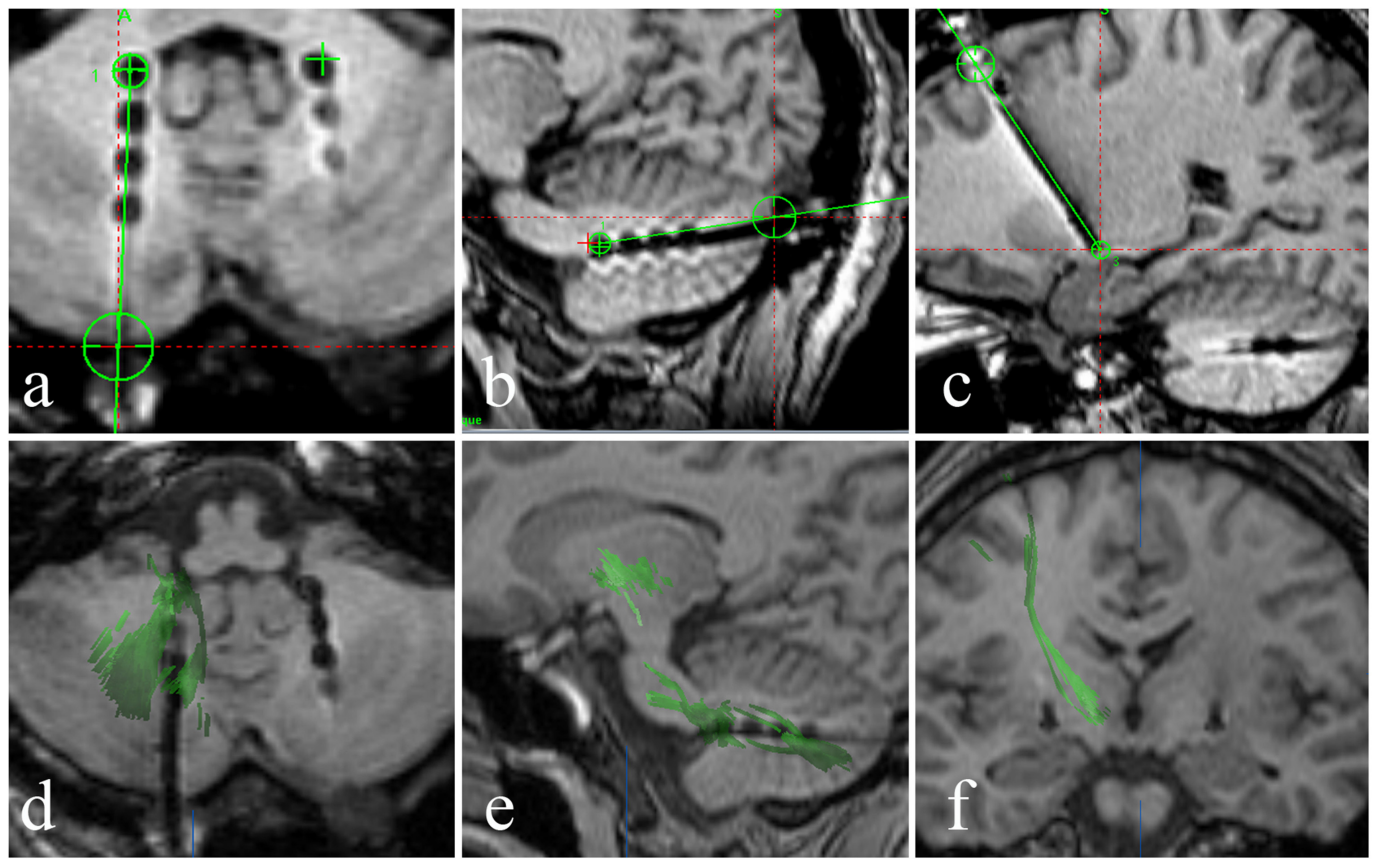
**(a–c)** A fused image of the intraoperative 3DT1-weighted sequence and the preoperative plan shows that the stimulation lead position and pathway (black dot and line) are consistent with the preoperative plan (green line), and the stimulation lead position is accurate. **(d, e)** Axial and sagittal diffusion tensor imaging shows the dentatorubrothalamic tract and the relationship between the dentatorubrothalamic tract and stimulation lead in the DN. **(f)** A coronal 3DT1 image showing the relationship between the pyramidal tract and the stimulation lead in the GPi.

The DN is visible in T2WI-FLAIR MRI (Figure 1). It is situated adjacent to the fourth ventricle, buried in white matter, and measures ~9–20 mm in width, 13–23 mm in length, and 7–20 mm in height. It has a unique shape and is oriented in a craniocaudal direction and from lateral to medial (8, 9, 11–13). With reference to previous studies (14, 15), we targeted the tip of each stimulation lead to the origin of the superior cerebellar peduncle such that the electrode contacted the anterior and upper third of the DN, a region related to motor function. In our patient, the anatomical target coordinates for DN stimulation were 15 mm lateral, 26 mm posterior, and 30 mm inferior to the midpoint of the anterior commissure-posterior commissure (ACPC) line on the left side, and 13 mm lateral, 28 mm posterior, and 31 mm inferior to the midpoint of the ACPC line on the right side. For the DN pathway, according to the DN position and the electrode covering the anterior and upper third of the DN, the entry point should be as close to the transverse sinus as possible (Figures 3a, b). For this patient, the trajectory of the “Ring” angle to the left and right DN was 4.6° and 8.8°, respectively. The “Arc” angle to the left and right DN was 82.2° and 92.4°, respectively. The posteroventral GPi is one of the most frequently used targets in Parkinson's disease DBS, and we did not target it here.

### 2.5. The surgical procedure

Under local anesthesia, the patient was placed in a stereotactic head frame (Leksell model G head frame) before stereotactic CT was performed. CT data were combined with the preoperative plan that was developed using MRI performed with a 3.0-T scanner (Siemens Espree). We first performed GPi DBS and then DN DBS. The operation was performed under general anesthesia with Remifen and propofol. The PINSL301 electrodes for DBS were placed in GPi (PINS Medical Co., Beijing, China) and the PINSL303 electrodes (quadripolar leads with 3 mm active contacts and 4 spacing) in DN (PINS Medical Co., Beijing, China). The leads were temporarily stimulated, and the wound was sutured. Intraoperative MRI (Siemens Espree, 1.5T) was then performed. We reconstructed the dentatorubrothalamic tract by fiber tracking to observe its relationship with the lead (BrainLab, Feldkirchen, Germany) (Figure 3).

### 2.6. The programming design

The severity of cerebellar ataxia and dystonia was assessed 1 month before surgery and then after surgery with a double-blind, randomized protocol in five phases: no stimulation, GPi, DN, or sham stimulation, and combined GPi and DN stimulation. To reduce the chance of infection, we shortened the stimulation test time as much as possible to 3 days for each phase. The acute stimulation test was performed using an external stimulation device (T901 Temporary stimulator, PINS Medical Co., Beijing, China). The principle of selecting stimulation parameters and contacts is to achieve the best effect without side effects.

## 3. Results

### 3.1. Postoperative efficacy of DBS of two targets

#### 3.1.1. Postoperative phase without stimulation

In most Parkinson's patients, after DBS surgery, especially for subthalamic nucleus DBS, there is a certain degree of “microlesioning”. For this patient, to eliminate the interference of “microlesioning”, no program control measures were taken 1–3 days after surgery. However, for this patient, we assessed various scores on the 3rd day. Most of the symptoms did not improve; only eye opening had a 25% improvement, and the heel-shin slide had a 33% improvement (Tables 1, 2).

#### 3.1.2. Bilateral posteroventral GPi stimulation

On postoperative day 4, temporary external stimulation was commenced for the GPi. The stimulation parameters were programmed to maximally alleviate the dystonia but not to produce stimulation-related side effects. The final GPi stimulus parameters were: R: contacts 1–, 3+, 90 μs, 185 Hz, and 3 V; L: contacts 5–, 7+, 90 μs, 185 Hz, and 3 V. The BFMDRS movement scores were evaluated on the 3rd day of GPi stimulation. There was a slight improvement of ~30% (from 40.5 to 28.5) in the eye, mouth, neck, and leg; however, there was no improvement in speech and swallowing, arm, or trunk. The BFMDRS disability and SARA scores improved by 12.5 and 4%, respectively (Table 2).

#### 3.1.3. Bilateral DN stimulation

On postoperative day 7, temporary external stimulation was initiated for the DN. The stimulation parameters were programmed to maximally alleviate the balance disorder and gait abnormality but not to produce stimulation-related side effects. The final DN stimulus parameters were: R: contacts 2–, 3+, 200 μs, 60 Hz, and 2 V; L: contacts 6–, 7+, 200 μs, 60 Hz, and 2 V. The SARA scores were evaluated on the third day of DN stimulation. There was a slight improvement of ~38% (from 24 to 15) in gait, stance, speech disturbance, nose-finger test, and heel-shin slide; however, there was no improvement in sitting, finger chase, or fast alternating hand movements. The BFMDRS disability and BFMDRS movement scores did not improve (Table 1).

#### 3.1.4. “Sham” stimulation

On postoperative day 10, the stimulation was stopped. Improved symptoms and signs returned to preoperative scores (Tables 1, 2).

#### 3.1.5. Bilateral posteroventral GPi + DN stimulation

On postoperative day 13, two targets were stimulated at the same time. The final evaluation showed that the total SARA scores improved by ~42% (from 24 to 14), the total BFMDRS movement scores improved by ~30% (from 40.5 to 28.5), and the total BFMDRS disability scores improved by ~12.5% (from 16 to 14). Although SARA and BFMDRS movement scores were partially improved, the patient's BFMDRS disability and activities of daily living scores barely improved; therefore, the patient was slightly anxious about the efficacy of the treatment, with a HAMA score change from 9 to 11. The cognitive function of the patient did not change (Tables 1–3).

### 3.2. Differences in ^18^F-FDG PET before and after stimulation

Preoperative ^18^F-FDG images showed hypometabolism in the bilateral cerebellar hemisphere (Figures 2a–c) and normal metabolism in the bilateral cerebral cortex (Figure 2d). The patient had a slight reaction to levodopa before surgery; therefore, we performed DAT PET/CT. The patient had normal DAT density in the bilateral caudate nucleus and putamen (Figure 2e). After 2 weeks of stimulation, the patient underwent a multimodal imaging approach, including ^18^F-FDG PET and 11C-β-CFT. Compared with the findings in preoperative images, ^18^F-FDG uptake significantly increased in the cerebellum, especially in the right cerebellar hemisphere (Figures 2a'–c'). There was no difference in ^18^F-FDG in the cerebral cortex (Figure 2d') or in DAT imaging (Figure 2e') before and after stimulation.

The brain MRI showed marked atrophy of the cerebellum and mild atrophy with enlargement of the fourth ventricle. The fused image of the intraoperative 3DT1-weighted sequence and the preoperative plan showed that the stimulation lead position and pathway were consistent with the preoperative plan and that the lead position was accurate (Figures 3a–c). We reconstructed the dentatorubrothalamic tract by fiber tracking. It can be seen that the lead is in close proximity to the fiber bundle passing through the DN (Figures 3d, e). The fiber bundle projects to the thalamus as the relay station and reaches the cerebral motor cortex (Figure 3f).

## 4. Discussion

### 4.1. Postoperative efficacy

Although we used a short-term stimulation schedule, this is the first report of SCA3/MJD treatment using multi-target DBS (DN and GPi). For this patient, two targets were stimulated at the same time. The final improvement was 42% in SARA, 30% in BFMDRS movement, and 12.5% in BFMDRS disability scores. Daily living was barely improved; therefore, because the patient had high expectations for the procedure, she was slightly anxious about the efficacy of the treatment. Only a few SCA3/MJD cases with DBS treatment have been reported, and all of them were single-target DBS (3–8). In 2015, Teixeira et al. reported a 50-year-old right-handed woman who underwent resection of a right acoustic neuroma, which was complicated by an ischemic injury of the right cerebellar hemisphere. She developed severe ataxia, mostly right-sided, that significantly impaired her daily activities, and mild bilateral hand and cervical dystonia. The patient underwent DBS of her (healthy) left DN. After 1 year, there was an improvement in tremor (37% reduction) and cerebellar ataxia from 25.5/40 to 17/40 (SARA score) on stimulation; however, there were no changes in dystonia after treatment (3). In 2018, Aupy et al. reported a 19-year-old man with a positive family history of SCA3/MJD, generalized dystonia, cerebellar ataxia, recurrent falls, and swallowing difficulties who underwent bilateral GPi implantation. Twelve months after surgery, the patient was able to stand up without help, and his swallowing was dramatically improved. However, his cerebellar ataxia did not improve (4). In 2019, Cury et al. reported a 31-year-old female with SCA3/MJD and refractory ataxia who underwent bilateral DN DBS. She showed improvements in tremor of ~30% and cerebellar ataxia of ~22% (SARA score). This was the first time the DN was targeted in an SCA3/MJD patient (5). In 2021, Cury et al. also reported a group of five SCA3/MJD patients treated with DN DBS. The effects on ataxia were numerically better in four out of five patients after active vs. sham stimulation. The composite SARA score did not change after comparing active to sham stimulation (*p* = 0.223) (6). The FTMRS score showed significant improvement after active stimulation vs. sham (*p* = 0.039), as did the patients' global impression of change (*p* = 0.038). The quality of life was not modified by stimulation (*p* = 0.337) (6). Our patient has both dystonia and cerebellar ataxia. According to previous reports, it is very difficult to solve both dystonia and cerebellar ataxia in SCA3/MJD with single-target stimulation (such as DN or GPi). We, therefore, adopted double-target stimulation: the DN to relieve cerebellar ataxia and tremor and the GPi to relieve dystonia. Our results show that stimulation of the GPi can relieve some symptoms of dystonia, such as tension in the eye, mouth, neck, and leg; however, the effects were poor for speech and swallowing, and arm and trunk. DN stimulation can relieve some symptoms of cerebellar ataxia, such as impaired gait, stance, nose-finger test, heel-shin slide, and speech disturbance; however, the effect on sitting, finger chase, and fast alternating hand movements was poor. Although there was some improvement in dystonia and cerebellar ataxia, the patient was not satisfied with the improved symptoms or their quality of life. This eventually led to the patient's request to remove the leads and abandon the implantation of the pulse generator.

### 4.2. ^18^F-FDG PET changes

The anatomical MR images of our patient revealed significant gray matter volume loss in the bilateral cerebellum and right cerebellar vermis, which is consistent with morphological imaging of SCA3/MJD (23–25).

Another finding was that, after DN stimulation, metabolism in the bilateral cerebellar hemispheres significantly changed, with metabolism significantly increasing compared with that before stimulation. This is the first demonstration of improvement of cerebellar hemisphere metabolism in an SCA3/MJD patient by stimulation of the DN and GPi. Local glucose metabolism and oxygen use are coupled with local brain activity, which indicates that *in vivo* measurement of regional glucose consumption by ^18^F-FDG PET provides an index of regional neuronal activity (26). Under physiological steady-state conditions, cerebral blood flow is tightly coupled to the level of cerebral oxygen and glucose consumption (27). In our patient, metabolism increased after cerebellar hemisphere stimulation increased cerebral blood flow, which is conducive to the recovery of neuron function. Relative decreases in ^18^F-FDG uptake in cerebellar hemispheres may reflect either impaired neuronal function caused by cellular pathology at that location or remote functional network changes caused by lesions elsewhere (28). It is uncertain whether the metabolic transition from low to high in our patient is stimulated by local neurons (DN DBS) or regulated by a remote functional network (GPi DBS). For our patient, we consider that cerebellar metabolism was promoted by DN DBS because DN DBS acts directly on the cerebellum; however, we cannot rule out an effect of GPi DBS.

Fukuda et al. (29) studied metabolic changes in the brain and cerebellum in Parkinson's disease patients treated with GPi DBS using ^18^F-FDG PET. They found that GPi DBS improved Unified Parkinson's Disease Rating Scale motor scores by 36% and significantly increased regional glucose metabolism in the premotor cortex ipsilateral to stimulation and in the cerebellum bilaterally. In addition, subthalamic nucleus DBS can change cerebellar metabolism (30–32). In our patient, diffusion tensor imaging fiber bundle dentatorubrothalamic tract reconstruction showed the fiber connection between the thalamus, pyramidal tract, motor cortex, and DN. GPi DBS may indirectly stimulate the cerebellum through the pallidothalamic tract and the dentatorubrothalamic tract. If GPi DBS can change the metabolism of the cerebellum, DBS is more likely to function by regulating the entire neural network rather than merely exciting or inhibiting certain nuclei.

Altogether, acute DN and GPi DBS lead to increased cerebellar metabolism, which is an encouraging finding and supports DBS treatment of SCA3/MJD.

### 4.3. Limitations

The stimulation test used external stimulation, and the duration of each group of tests was short, only 3 days, especially for the GPi; however, prolonged stimulation can play a regulatory role. Although this was a prospective, double-blind study, it included only one case.

## 5. Conclusion

Multi-target acute stimulation (DN DBS and GPi DBS) in SCA3/MJD can mildly improve cerebellar ataxia and dystonia. Acute stimulation also increased cerebellar metabolism. Long-term efficacy and brain metabolic changes after SCA3/MJD DBS need further study.

## Data availability statement

The original contributions presented in the study are included in the article/Supplementary material, further inquiries can be directed to the corresponding authors.

## Ethics statement

The studies involving human participants were reviewed and approved by the Ethics Committee of the Chinese People's Liberation Army General Hospital. The patients/participants provided their written informed consent to participate in this study. Written informed consent was obtained from the individual(s) for the publication of any potentially identifiable images or data included in this article.

## Institutional review board statement

This study was conducted in accordance with the Declaration of Helsinki and was approved by the Institutional Review Board (or Ethics Committee) of the Chinese People's Liberation Army General Hospital (S2019–268-02; October 1, 2019) (Chinese Clinical Trials: 2100045363).

## Author contributions

Conceptualization and original draft preparation: ZC. Methodology and data curation: JW. Imaging materials: YL, XLi, and XLo. Nuclear medicine image data: YC and RW. All authors have read and agreed to the published version of the manuscript.

## Supplementary material

Supplementary Video S1 This patient has degenerative ataxia and walks with poor balance, no normal standing posture, and loss of independent walking ability, with a wide base, and irregular steps. There is also an occasional lurch.

Supplementary Video S2 The patient's movement of straight walking and turn-back must be supported by someone, with the patient's center of gravity on her upper body, and relying on a forward leaning posture. The hands of the helper are used to maintain the balance of the patient's body.

Supplementary Video S3 When the stimulator is turned on, the patient feels an increase in muscle strength in both lower limbs. Although there is still a significant balance disorder, she is able to stand and walk alone. But the turning motion is still slow and not yet fully completed.

Supplementary Video S4 When the stimulator is turned on, there is also an occasional lurch. The patient is able to walk alone with their left hand supporting the wall.
